# Optimization of concentrations of different n-3PUFAs on antioxidant capacity in mouse hepatocytes

**DOI:** 10.1186/s12944-024-02202-0

**Published:** 2024-07-09

**Authors:** Shuting Wang, Huasong Bai, Tong Liu, Jiayi Yang, Zhanzhong Wang

**Affiliations:** 1 Nourse Science Centre for Pet Nutrition, Wuhu, 241200 China; 2 School of Chemical Engineering and Technology, Tianjin University, Tianjin, 300072 China

**Keywords:** N-3 PUFAs, Antioxidant capacity, Box-Behnken design, Response surface methodology

## Abstract

Omega-3 polyunsaturated fatty acids (n-3 PUFAs), mainly including α-linolenic acid (ALA), eicosapentaenoic acid (EPA), and docosahexaenoic acid (DHA), possess antioxidant properties and play a crucial role in growth and development. However, the combined effects of ALA, EPA, and DHA at different concentrations have rarely been reported. This work explored the effects of EPA, ALA, and DHA on the viability and antioxidant capacity of mouse hepatocytes, with the objective of enhancing the antioxidant capacity. Within the appropriate concentration range, cell viability and the activity of glutathione S-transferase, superoxide dismutase, and catalase were increased, while the oxidation products of malondialdehyde and the level of intracellular reactive oxygen species were obviously reduced. Thus, oxidative stress was relieved, and cellular antioxidant levels were improved. Finally, response surface optimization was carried out for EPA, ALA, and DHA, and the model was established. The antioxidant capacity of the cells was highest at EPA, ALA, and DHA concentrations of 145.46, 405.05, and 551.52 µM, respectively. These findings lay the foundation for further exploration of the interactive mechanisms of n-3 PUFAs in the body, as well as their applications in nutraceutical food.

**Supplementary Information:**

The online version contains supplementary material available at 10.1186/s12944-024-02202-0.

## Introduction

Omega-3 polyunsaturated fatty acids (n-3 PUFAs) are polyunsaturated fatty acids that are key components of human and animal tissues. As precursor pools, n-3 PUFAs can be converted to bioactive derivatives [1]. n-3 PUFAs alter blood vessels and carcinogenic biomarkers and have antioxidant, anti-inflammatory, and lipid-lowering effects. Thus, they reduce the risk of various diseases, including cancer [2–4].

n-3 PUFAs are mainly composed of α-linolenic acid (18:3 n-3, ALA), eicosapentaenoic acid (20:5 n-3, EPA), and docosahexaenoic acid (22:6 n-3, DHA) [5]. Relevant studies have found that they can regulate some important biological antioxidant pathways and have different physiological functions [6–8]. ALA contributes to the synthesis of neurotransmitters, which is crucial for the transmission of nerve impulses and the maintenance of cognitive functions. Additionally, ALA can help reduce the formation of blood clots, alleviate oxidative stress, and mitigate inflammatory responses. ALA also plays a significant role in regulating specific physiological metabolism [9]. Wang et al. found that appropriate amounts of ALA could reduce peroxidative damage by improving antioxidant oxidase activity and scavenging free radicals [10]. However, excess ALA can promote mitochondrial fatty acid oxidation [11, 12]. EPA and DHA can enhance the fluidity and flexibility of cell membrane structures, thereby improving cell signaling and nutrient transport. EPA is beneficial for maintaining the regular function and maturation of the brain and retina and can also reduce oxidative stress and inflammation [13]. Some studies [14–16] showed that dietary supplementation with EPA could improve the activity of antioxidant enzymes. Other studies [17–19] found that EPA could reduce oxidative stress by improving mitochondrial function. DHA can affect neuronal signal transduction, which is crucial for promoting brain development, mitigating oxidative stress and inflammation, and is beneficial to vision development [20, 21]. Mora et al. [22] confirmed that DHA could improve the antioxidant capacity of cells by increasing the glutathione (GSH) content and the activity of superoxide dismutase (SOD). Guillot et al. [23] found that DHA could positively influence platelet function and redox status, potentially reducing the risk of heart-related incidents among men in good health. Calzada et al. [24] concluded that consuming 200–800 milligrams of DHA daily might offer low-density lipoprotein (LDL) antioxidant benefits. Higher doses did not provide additional benefits and might not be necessary for achieving the desired effects on LDL redox status. However, the effects of higher doses of DHA appear to be more complex, with indications of both beneficial and adverse effects, depending on the dosage. In addition, DHA and EPA enhance antioxidant defenses through different pathways [25]. However, in human pancreatic cancer cells, excessive EPA and DHA can increase reactive oxygen species (ROS) levels, promote apoptosis, and reduce the antioxidant capacity of cells [26, 27]. Therefore, ALA, DHA, and EPA each play significant roles as biological antioxidants, but their interactive pathways and mechanisms could differ.

Some elongation and desaturation processes can enable the partial conversion of ALA to EPA and DHA in animals, but the conversion rate is limited. Consequently, dietary supplementation is often necessary to ensure adequate supplies of ALA, EPA, and DHA, which are crucial for various physiological functions and health outcomes. Some studies have reported the EPA and DHA concentrations in food. The research suggested that under the condition of a high-fat diet, the antioxidant capacity was best when the DHA/EPA ratio was 2:1 [28]. A study by Wang et al. projected the ideal ratio of DHA to EPA in mud crabs to range from 23:10 to 178:25 [29]. However, research on the optimal concentrations of EPA, ALA, and DHA for human health has rarely been reported [30]. Therefore, studies on the effect of n-3 PUFAs on the antioxidant capacity of hepatocytes and their optimal supplemental concentrations are needed to improve the bioavailability of n-3 PUFAs.

In this work, the impact of EPA, ALA, and DHA on the viability of mouse hepatocytes was first investigated using cell experiments to determine the treatment time and concentration range for use in subsequent studies, and then the impact of EPA, ALA, and DHA on the antioxidant capacity of the cells was explored. The contents of ROS and malondialdehyde (MDA), as well as the activities of the antioxidant enzymes glutathione S-transferase (GST), SOD, and catalase (CAT) were assessed, and the antioxidant mechanism was analyzed. Finally, response surface optimization was conducted to analyze the interactions between EPA, ALA, and DHA and determine their optimal concentrations, aiming to provide a foundation for the in-depth study of the mechanism of n-3 PUFAs on living beings, including animals, especially pets, and their applications in nutraceutical food.

## Materials and methods

### Materials

ALA (99% mass fraction purity) was purchased from Aladdin Biochemical Technology Co., Ltd., Shanghai, China. EPA was purchased from Shanghai D&B Biological Science and Technology Co., Ltd., Shanghai, China, and DHA was purchased from Bide Pharmatech Co., Ltd., Shanghai, China. NCTC 1469 mouse hepatocyte**s** were kindly provided by Procell Life Science & Technology Co., Ltd., Wuhan, China. Roswell Park Memorial Institute (RPMI) medium 1640 basic (1×) and fetal bovine serum (FBS) were purchased from Gibco, Grand Island, NY, USA. Phosphate-buffered saline (PBS), penicillin-streptomycin liquid (100X), bovine serum albumin (BSA), the Cell Counting Kit-8 (CCK-8), the MDA content assay kit, GST, SOD, and CAT activity assay kits, the Bicinchoninic Acid Assay (BCA) Protein Concentration Determination Kit, and the ROS assay kit were obtained from Solarbio Science and Technology Co., Ltd., Beijing, China.

### Methods

#### Exploration of appropriate time and fatty acid concentrations

##### NCTC 1469 cell tissue culture

NCTC 1469 cells are an epithelial cell line derived from the liver of a newborn mouse. The cells were kept at 37 ºC, 95% air, and 5% CO_2_ in RPMI 1640 basic medium supplemented with 10% FBS, 100 U/mL penicillin, and 100 mg/mL streptomycin. Experiments were performed with cells in good growing condition. These cells are characterized by their ability to grow both adherently and in suspension, making them versatile for various research applications, including studies on cell growth, metabolism, and responses to different experimental treatments.

### n-3 PUFA supplementation

NCTC 1469 cells were incubated with n-3 PUFAs. n-3 PUFAs at different concentrations were dissolved in absolute ethanol and complexed to BSA. The final concentrations of absolute ethanol and BSA were 0.05% and 0.5%, respectively, of the serum-free culture medium in the experimental group. No n-3 PUFAs were added to the control group. The maximum concentration range was 0–200 µM for EPA, 0–600 µM for ALA, and 0–700 µM for DHA. The ratio of n-3 PUFAs to BSA was maintained at a level that allowed for effective binding, not exceeding 2:1, to ensure the proper complexation of PUFAs with albumin. The cells were treated with individual n-3 PUFAs.

### Cell viability

The cells were diluted with complete culture medium and seeded in 96-well plates at 100 µL per well, reaching 80% confluency after 24 h of culture. n-3 PUFAs were added to the experimental and control groups at different concentrations. Six wells of cells were treated with each n-3 PUFA concentration. After adding CCK-8, the cells were incubated for 3 h at 37 ℃. Absorbance was measured at 450 nm using a microplate reader (Thermo Scientific Fluoroskan, Thermo Fisher Scientific, Waltham, MA, USA).

### Observation of cell morphology

After the cells were cultured for certain periods (24, 48, and 72 h), the morphology of each group of cells was observed under an inverted fluorescence microscope, (UOP Photoelectric Technology Co., Ltd., Chongqing, China).

#### Single-factor design for the antioxidant capacity of the n-3 PUFAs

##### ROS levels

The ROS fluorescent probe method was used to detect cellular radical levels. After adding the n-3 PUFAs for 24 h to the cells, 100 µL of 2’,7’-dichlorodihydrofluorescein diacetate (DCFH-DA) diluted 1:1000 with phenol red serum-free culture medium was added to each well. After 30 min, the liquid in the wells was discarded, and the cells were washed three times with serum-free culture medium. After the probe was loaded, the fluorescence intensity was measured. The fluorescence intensity of the cells was observed and photographed using an inverted fluorescence microscope (UOP Photoelectric Technology Co., Ltd.).

### MDA content

After the cells were digested with pancreatic enzymes, they were centrifuged, and the supernatant was discarded. After adding the extraction working solution provided in the kit, the cells were disrupted on ice with an ultrasonic disruptor and then centrifuged. The suspension was then added to the corresponding reagents in the kit. After centrifuging, absorbance was measured at 532 and 600 nm.

### GST activity

After the cells were digested with pancreatic enzymes, they were centrifuged, and the supernatant was discarded. After adding the extraction working solution provided in the kit, the cells were disrupted on ice with an ultrasonic disruptor and then centrifuged. The suspension was added to the corresponding reagent in the kit. Finally, absorbance was measured at 340 nm and GST actively was calculated according to the formula given by the kit manufacturer and protein concentration.

### SOD activity

The cell lysis and centrifugation procedures were the same as those in the GST activity assay. SOD activity and protein concentrations were measured in the cell suspension. The suspension was added to the prepared working solution in the kit, and the absorbance of the assay and control tubes was measured at 560 nm using a microplate reader (Thermo Scientific Fluoroskan) after 30 min of incubation in a water bath at 37ºC. Intracellular SOD activity was calculated according to the formula given by the kit manufacturer and protein concentration.

### CAT activity

The cell lysis and centrifugation procedures were the same as those in the GST activity assay. CAT activity and protein concentration were measured in the cell suspension. The suspension was added to the prepared working solution in the kit, and the absorbance was measured at 240 and 240 nm. Intracellular CAT activity was calculated using the formula given by the kit manufacturer and protein concentration.

### Protein concentration determination

After adding the supernatant, 200 µL of prepared BCA working solution was added, and the absorbance was measured at 562 nm. Finally, the protein concentration of the sample was calculated.

### Optimization of n-3 PUFA concentrations for antioxidant effects

According to the design principle of Box-Benhnken center combination, comprehensive univariate test results, the independent variables in this study were EPA, ALA, and DHA concentrations, and the response values were ROS, GST activity, and MDA content. The response surface optimization test of three factors and three levels was carried out. The experimental design and independent variable levels are shown in Table 1. It should be noted that mixtures of the three n-3 PUFAs at different concentrations were added.

**Table 1:** Factors and levels in the response surface test obtained by the single-factor experiment

Levels	Factors		
	EPA (µM)	ALA (µM)	DHA (µM)
-1	100	300	400
0	150	400	550
1	200	500	700

### Validation and determination of optimal n-3 PUFA concentrations for antioxidant effects

The predicted data were compared with the experimental data, and the n-3 PUFA concentrations with the strongest antioxidant effects in mouse hepatocytes were obtained through validation experiments.

### Statistical analysis

Each experiment was conducted in triplicate, and all outcomes are shown as the mean ± standard deviation. Graphs were drawn using Origin 2020. The data were evaluated by analysis of variance (ANOVA) using the Statistical Product and Service Solutions (SPSS) 18.0 software package. Multiple regression analysis was performed using Minitab Statistical Software 21.

## RESULTS

### Effects of different n-3 PUFAs on cell proliferation

#### Cell viability assay

**Fig. 1:**
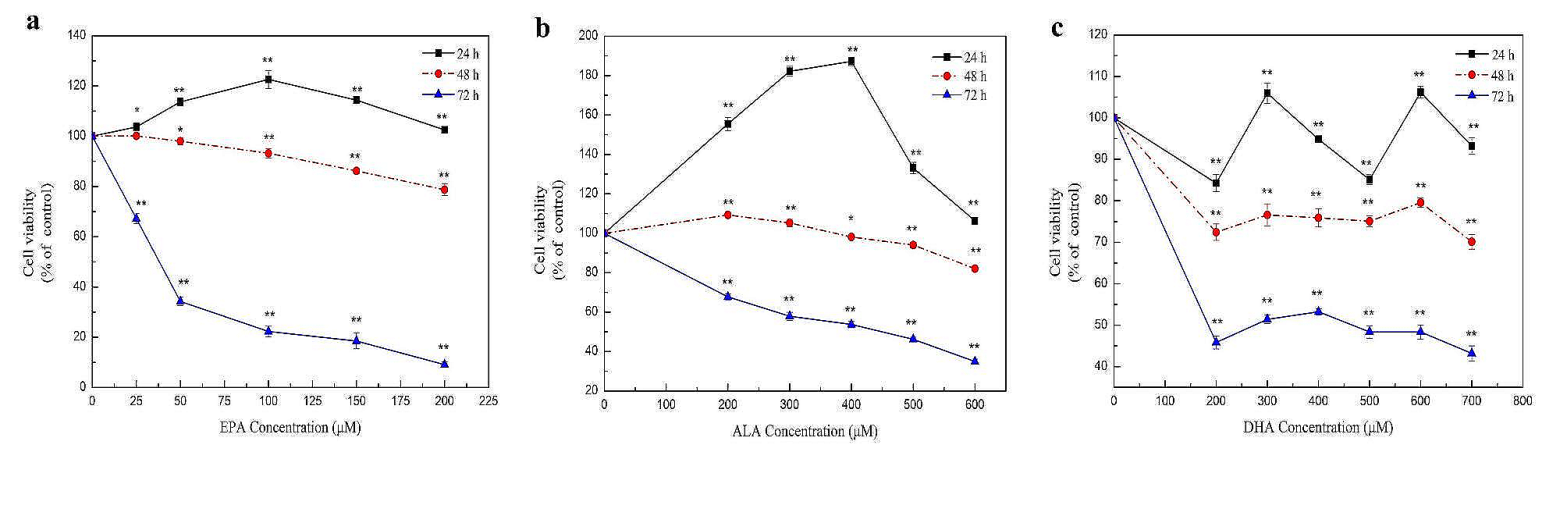
Effect of different concentrations of n-3 PUFAs on cell viability at different treatment times. **(a)** The effect of 0–200 µM EPA on cell viability at 24, 48, and 72 h. **(b)** The effect of 0–600 µM ALA on cell viability at 24, 48, and 72 h. **(c)** The effect of 0–700 µM DHA on cell viability at 24, 48, and 72 h. In the follow-up experiment, the conditions with little effect on cell viability were selected, namely EPA 0–200 µM, ALA 0–600 µM, and DHA 0–700 µM for 24 h. Significant at *P < 0.05, **P < 0.01

Cell viability was assayed to determine the effect of different EPA, ALA, and DHA concentrations on cell proliferation. In vitro experiments were conducted using NCTC 1469 mouse hepatocytes. As shown in Fig. 1a, all concentrations of n-3 PUFAs had significant effects on cell viability. After adding EPA for 24 h, the results showed a trend of first increasing and then decreasing, and cell viability was the highest after 24 h. Cell viability decreased slightly to about 80% after 48 h. With increases in treatment concentration, cell viability decreased sharply to about 10% after 72 h. After treatment with 50 µM EPA, most of the cells died. Similarly, Fig. 1b shows that cell viability increased significantly after treatment with 200–600 µM ALA for 24 h, which was greater than that after EPA and DHA treatment and cell viability was the highest after treatment with 400 µM ALA. After treatment for 48 h, ALA had little effect on cell viability. Cell viability was reduced to less than half after 72 h of treatment with 600 µM ALA. Figure 1c shows that cell viability fluctuated after adding DHA for 24 h. Similarly, cell viability decreased significantly after treatment for 24 h and 48 h, to about 80% and 40%, respectively. Since this work mainly investigated the effects on antioxidant capacity, EPA, ALA, and DHA concentrations and treatment times with small decreases in cell viability were selected for subsequent experiments, namely 0–200 µM EPA, 0–600 µM ALA, and 0–700 µM DHA for 24 h.

#### Observation of cell morphology

**Fig. 2:**
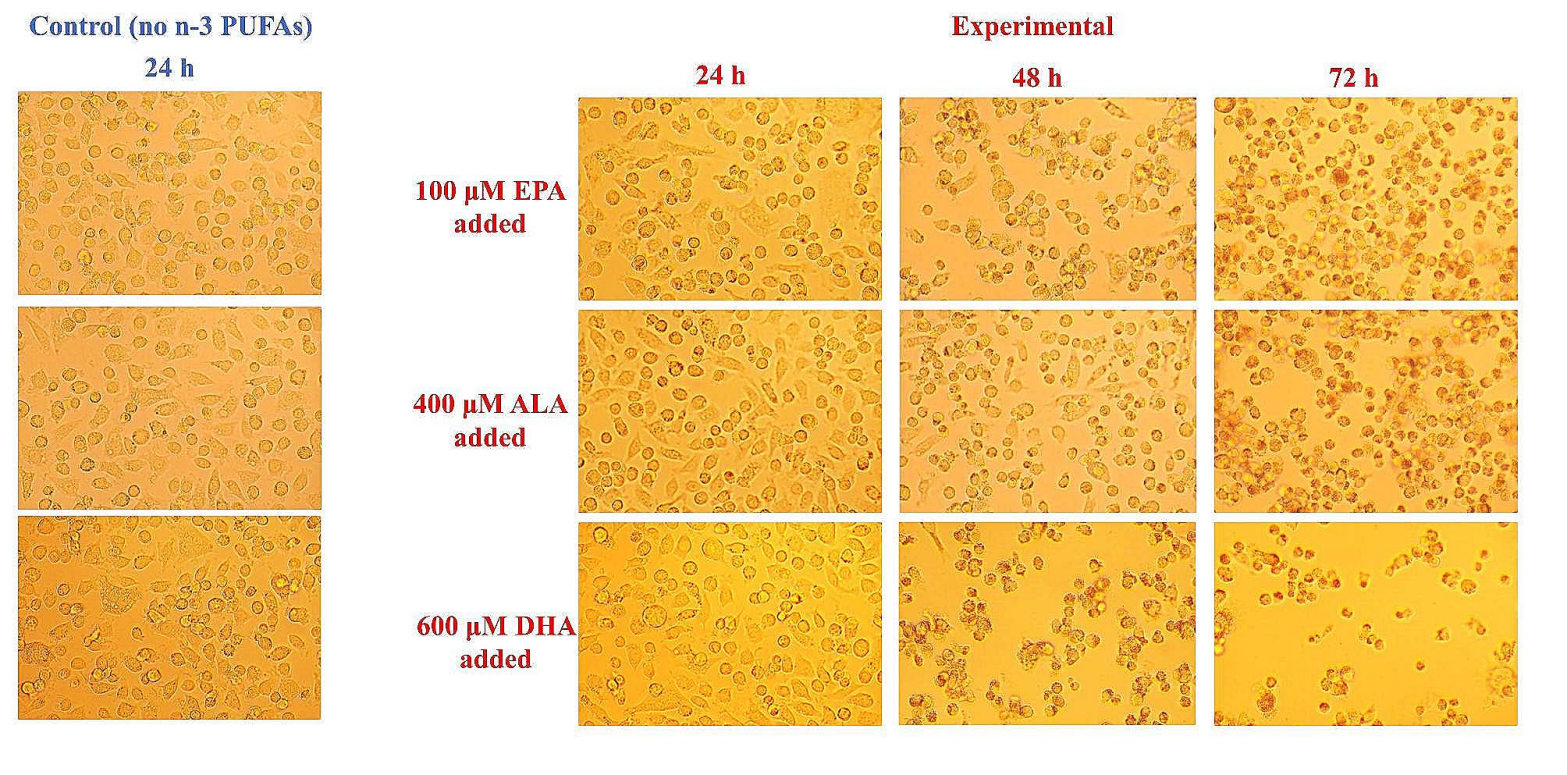
Changes in cell morphology after treatment with different concentrations of n-3 PUFAs for different times. The control group was not treated with n-3 PUFAs for 24 h. The experimental group was treated with 100 µM EPA, 400 µM ALA, and 600 µM DHA for 24, 48, and 72 h, respectively. The images are 400x magnifications. After treatment for 24 h, there was no significant difference in cell morphology between treated and control cells, and 24 h was selected as the treatment time for the follow-up experiments. Significant at *P < 0.05, **P < 0.01

The changes in cell morphology were observed under an inverted microscope (UOP Photoelectric Technology Co., Ltd., Chongqing, China) (Fig. 2). After 24 h of treatment with EPA, ALA, and DHA, the cell morphology was not significantly different from that of normal cells. Cells adhered to the surface of the plate, and the growth conditions were good. The cell shape was full, their outlines were clear, and the arrangement was close. With increases in time, the cells began to grow together after 48 h, and the cell morphology changed significantly. After 72 h, the edges of the cells were not clear, the clumping of cells was more common, some suspended dead cells appeared, and some cells died. Therefore, a treatment time of 24 h was selected for the follow-up experiments.

### Antioxidant effects of n-3 PUFAs on cells

#### Determination of ROS levels

**Fig. 3:**
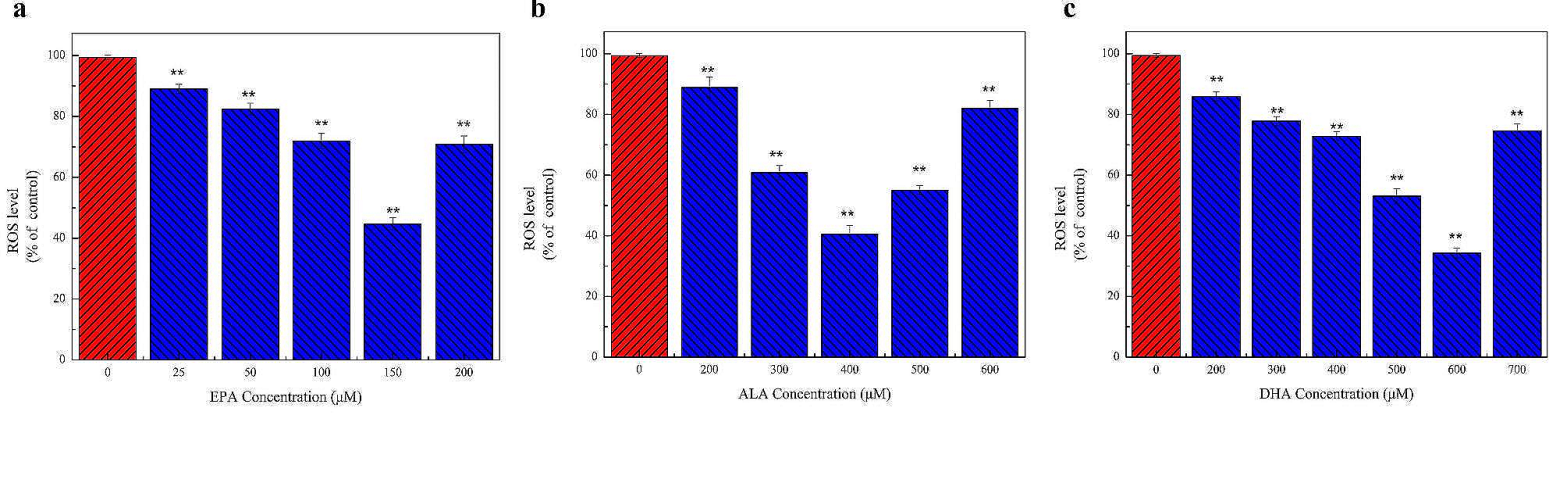
Effects of different concentrations of n-3 PUFAs on ROS levels in cells. The ROS level showed the effect of n-3 PUFAs on cellular antioxidant capacity. **a** The effect of 0–200 µM EPA treatment for 24 h on intracellular ROS levels. **b** The effect of 0–600 µM ALA treatment for 24 h on intracellular ROS levels. **c** The effect of 0–700 µM DHA treatment for 24 h on intracellular ROS levels. The results showed that appropriate concentrations of EPA, DHA, and ALA could reduce ROS levels and enhance the antioxidant capacity of the cells. *Significant at *P* < 0.05, **P < 0.01.

ROS is produced by mitochondria in cells during normal metabolic processes. An overabundance of ROS within cells can trigger oxidative stress, which can harm cells and result in the peroxidation of lipids within the liver membrane. As shown in Fig. 3a, ROS levels decreased in cells treated with 0–150 µM EPA for 24 h and increased at 200 µM EPA. Treatment with 150 µM EPA decreased ROS levels by about 60%. Figure 3b and c show that ROS levels in cells treated with increasing concentrations of ALA, DHA, and EPA decreased and then increased. ROS levels were the lowest at an ALA concentration of 400 µM and a DHA concentration of 600 µM.

Figure 3 shows ROS fluorescence imaging of cells treated with 150 µM EPA, 400 µM ALA, and 600 µM DHA for 24 h. The range of green fluorescence in the experimental group decreased and darkened, and the fluorescence intensity decreased significantly, indicating that ROS levels decreased significantly, and the antioxidant capacity of the cells increased

**Fig. 4:**
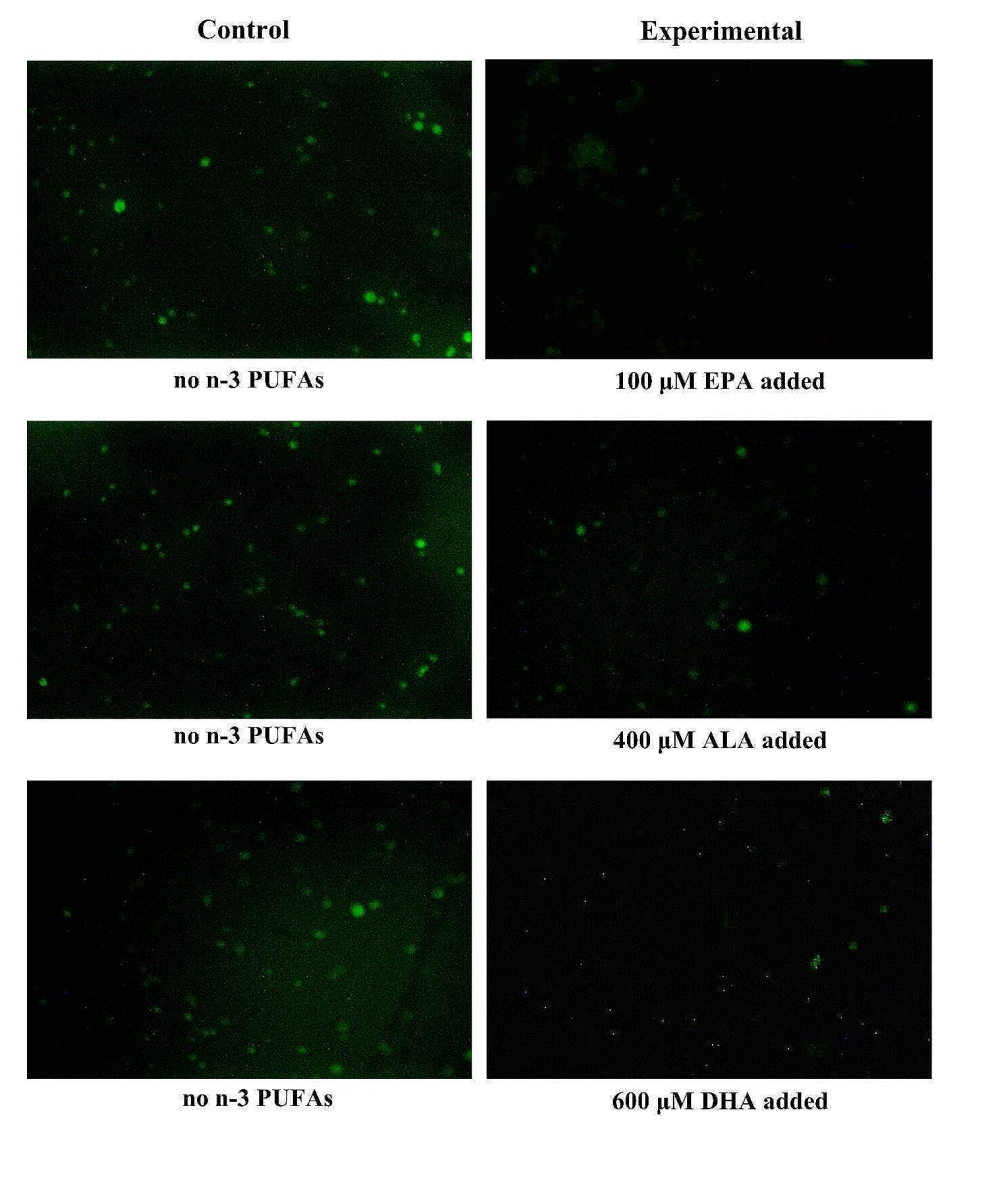
ROS fluorescence images of cells after adding different concentrations of n-3 PUFAs for 24 h. The control group was untreated for 24 h, and the experimental group was treated with 100 µM EPA, 400 µM ALA, and 600 µM DHA for 24 h. Images are 400x magnifications. ROS levels decreased significantly in the experimental group, and the antioxidant capacity of the cells increased. Significant at *P < 0.05, **P < 0.01

#### Determination of MDA content

**Fig. 5:**
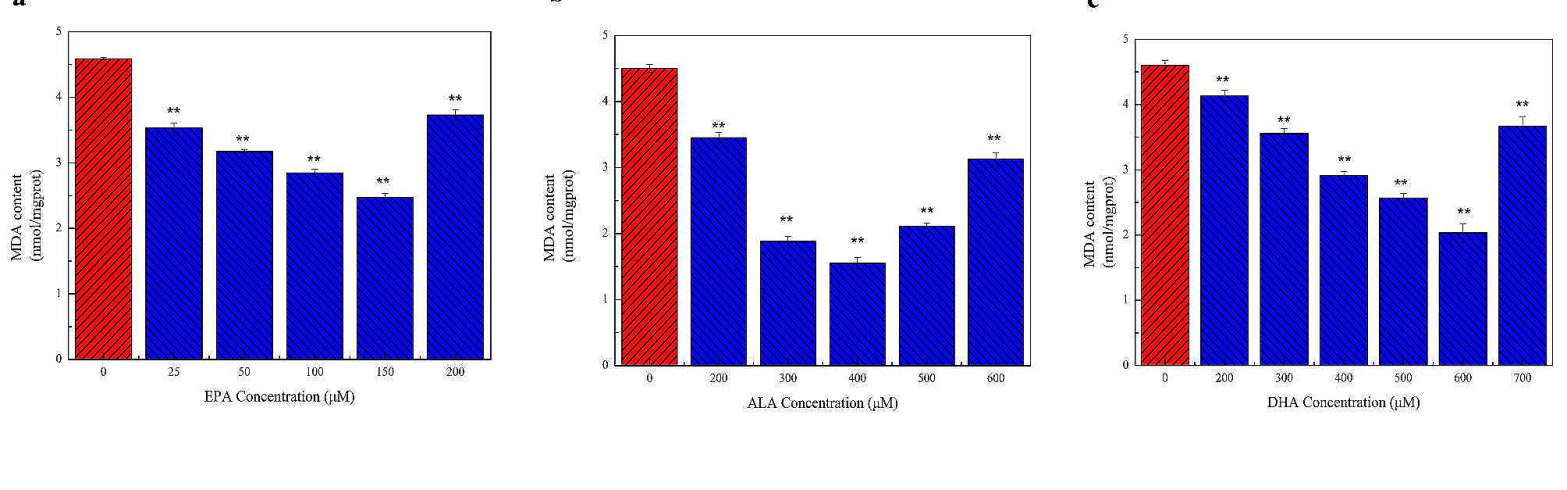
Effects of different concentrations of n-3 PUFAs on MDA content in the cells. The effect of n-3 PUFAs on the cellular antioxidant capacity was demonstrated by MDA content. **a** The effect of 0–200 µM EPA treatment for 24 h on intracellular MDA content. **b** The effect of 0–600 µM ALA treatment for 24 h on intracellular MDA content. **c** The effect of 0–700 µM DHA treatment for 24 h on intracellular MDA content. The results showed that appropriate concentrations of EPA, DHA, and ALA could reduce the MDA content and enhance the antioxidant capacity of the cells. Significant at *P < 0.05, **P < 0.01

Treatment with 0–150 µM EPA decreased the intracellular MDA content, whereas 200 µM EPA increased the content (Fig. 5a). At a treatment concentration of 150 µM, the intracellular MDA content decreased by about 45%. As shown in Fig. 5b and c, similar results were obtained with EPA. Within a certain concentration range, the intracellular MDA content first decreased and then increased with increases in ALA and DHA concentrations. The content of MDA and the degree of oxidative damage was the lowest at 400 µM ALA and 600 µM DHA.

#### Determination of GST activity

**Fig. 6:**
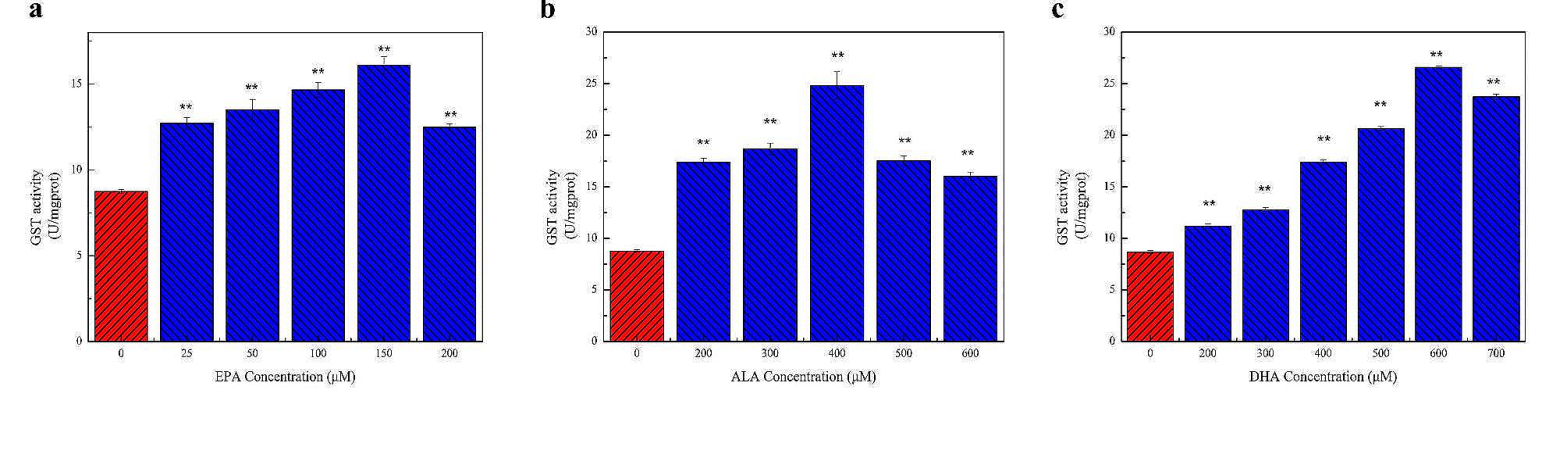
Effect of different concentrations of n-3 PUFAs on GST activity in the cells. The effect of n-3 PUFAs on cellular antioxidant capacity was demonstrated by GST activity. **a** The effect of 0–200 µM EPA treatment for 24 h on intracellular GST activity. **b** The effect of 0–600 µM ALA treatment for 24 h on intracellular GST activity. **c** The effect of 0–700 µM DHA treatment for 24 h on intracellular GST activity. The results showed that appropriate concentrations of EPA, DHA, and ALA could decrease GST activity and enhance the antioxidant capacity of the cells. Significant at *P < 0.05, **P < 0.01

The results described in Fig. 6a show that the intracellular GST activity of cells treated with EPA changed significantly compared with the control group. With increases in EPA concentrations, GST activity first increased and then decreased. The intracellular GST activity was the highest at 150 µM EPA, increasing by about 50%. At 200 µM, the GST activity decreased. Similarly, Fig. 6b and c show that GST activity first increased and then decreased in cells treated with ALA and DHA and reached the highest activity at 400 µM ALA and 600 µM DHA.

### Determination of SOD and CAT activity

**Fig. 7:**
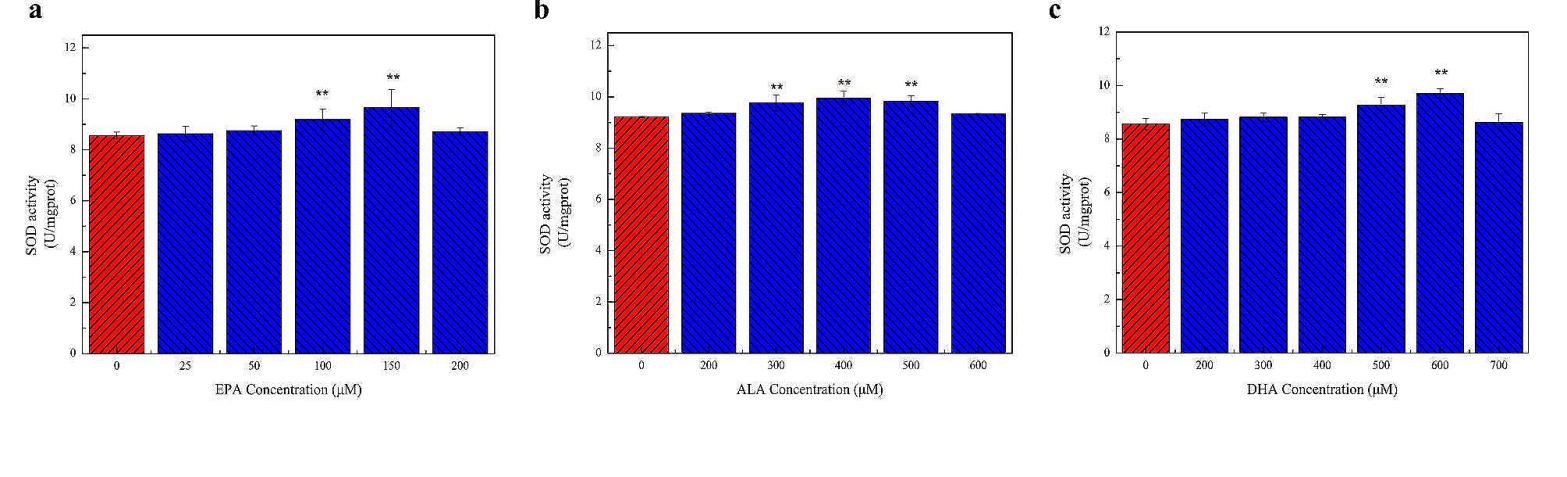
Effects of different concentrations of n-3 PUFAs on SOD activity in cells. SOD activity showed the effect of n-3 PUFAs on cellular antioxidant capacity. **a** The effect of 0–200 µM EPA treatment for 24 h on SOD activity in cells. **b** The effect of 0–600 µM ALA treatment for 24 h on SOD activity in cells. **c** The effect of 0–700 µM DHA treatment for 24 h on SOD activity in cells. The results showed that appropriate concentrations of EPA, DHA, and ALA could reduce SOD activity and enhance the antioxidant capacity of the cells. Significant at *P < 0.05, **P < 0.01

SOD is an antioxidant very important for maintaining the oxidant and antioxidant balance in cells. SOD activity increased significantly in cells treated with 100 and 150 µM EPA but did not change significantly at other concentrations (Fig. 7a). Similarly, 300–500 µM ALA significantly increased SOD activity, while changes in SOD activity were not obvious in cells treated with other concentrations of ALA (Fig. 7b and c). Treatment with 500 and 600 µM DHA significantly increased SOD activity, but no significant change was observed at other concentrations. Consistent with these findings, SOD activity was highest in cells treated with 150 µM EPA, 400 µM ALA, and 600 µM DHA.

**Fig. 8:**
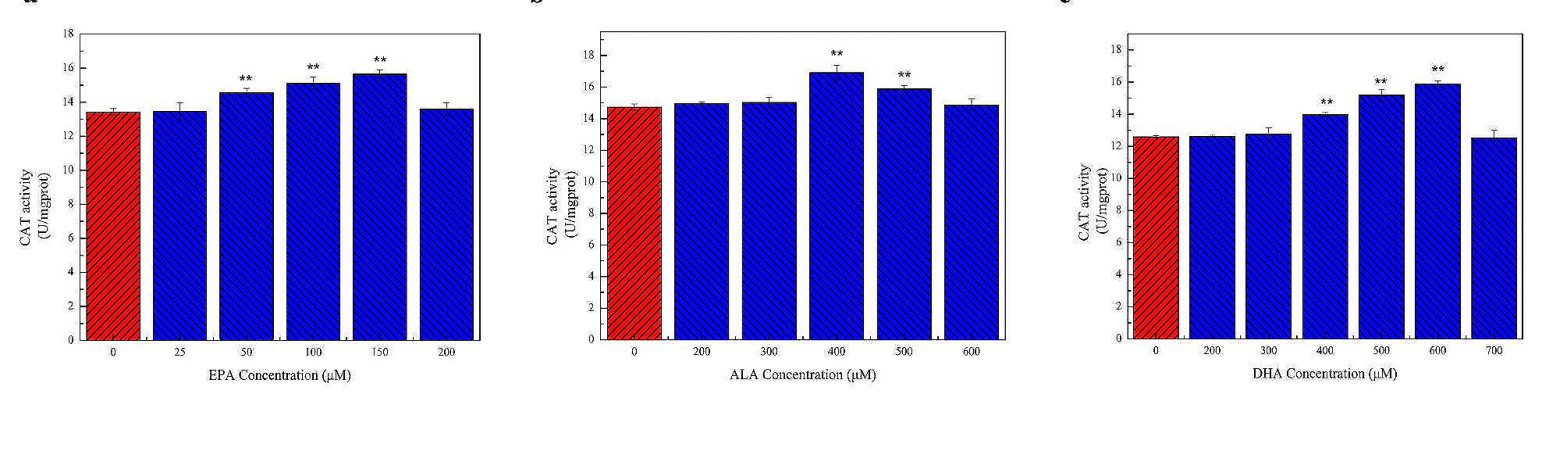
Effects of different concentrations of n-3 PUFAs on CAT activity in cells. CAT activity showed the effects of n-3 PUFAs on cellular antioxidant capacity. **a** The effect of 0–200 µM EPA treatment for 24 h on CAT activity in cells. **b** The effect of 0–600 µM ALA treatment for 24 h on CAT activity in cells. **c** The effect of 0–700 µM DHA treatment for 24 h on CAT activity in cells. The results showed that appropriate concentrations of EPA, DHA, and ALA could reduce CAT activity and enhance the antioxidant capacity of the cells. Significant at *P < 0.05, **P < 0.01

Treatment of cells with 50–150 µM EPA, but not other concentrations, significantly increased CAT activity (Fig. 8a). Similarly, intracellular CAT activity was significantly increased in cells treated with 400 and 500 µM ALA, whereas CAT activity was not significantly changed by treatment with other ALA concentrations (Fig. 8b and c). CAT activity was significantly increased in cells treated with 400–600 µM DHA, but no significant change was observed at other concentrations. Consistent with the SOD activity, intracellular CAT activity was the highest in cells treated with 150 µM EPA, 400 µM ALA, and 600 µM DHA.

### Response surface method to optimize the test design

The response surface method can determine the approximate relationship between a set of independent variables (EPA, ALA, and DHA concentrations) and responses and optimize the parameters to obtain the ideal response [31]. According to the design principle of Box-Benhnken center combination, the antioxidant capacity of mouse hepatocytes was optimized under the following conditions: 100–200 µM EPA, 300–500 µM ALA, and 400–700 µM DHA. ROS levels, GST activity, and MDA content all changed significantly within a certain concentration range, whereas SOD and CAT activity did not. Therefore, with EPA, ALA, and DHA as independent variables, ROS levels, MDA content, and GST activity were used as the response values. Thus, the response surface optimization experiment was conducted with three variables at three different levels. The experimental design and results are shown in Supporting Information Table S1.

## Model establishment and variance analysis

The results in Supporting Information Tables S2, S3, and S4 are fitted by quadratic multiple regression using response surface analysis software. Model equations were obtained using ROS levels (Y1), MDA content (Y2), and GST activity (Y3) as response values and EPA (A), ALA (B), and DHA (C) concentrations as independent variables.

1 $${Y}_{1}=16.867-0.557B+0.590AC+1.839{A}^{2}+8.127{B}^{2}+4.759{C}^{2}$$

2 $${Y}_{2}=0.844-0.054B+0.072AC+0.538{B}^{2}+0.287{C}^{2}$$

3 $${Y}_{3}=32.730+0.589B-0.589AC-1.557{A}^{2}-6.013{B}^{2}-3.398{C}^{2}$$

The determination coefficient, denoted as R^2^, serves as a metric to assess the degree of alignment. The R^2^ values were more than 0.99, and the adjustment coefficient (R^2^_Adj_) was more than 0.97 and could be used to predict the test results. The *P*-values for all three models were below the threshold of 0.01. Moreover, the *P*-values of the missing fitting terms of the three models were all greater than 0.05.

The results indicated that when ROS levels, MDA content, and GST activity were used as response values, ALA×ALA and DHA×DHA reached extremely significant levels (*P* < 0.01) while ALA and EPA×DHA reached very significant levels, and for ROS levels and GST activity, EPA×EPA reached a very significant level (*P* < 0.05). According to the *F* value in the models, the impact of various factors on ROS levels, MDA content, and GST activity showed that ALA exerted the most significant effect, followed by EPA and then DHA, indicating that ALA’s influence surpassed that of both EPA and DHA.

### Response surface analysis

The contort-line and response surface maps of the interactive effects of n-3 PUFA concentrations on ROS levels, MDA content, and GST activity are shown in Supporting Information Figures S1 and S2. As can be seen from Supporting Information Figures S1a-c and f-i, the contour lines were ellipses, indicating a significant synergistic enhancement effect between the various factors. Specifically, EPA, ALA, and DHA had significant interactions with ROS levels and GST activity, while ALA and DHA had significant interactions with MDA content. As shown in Supporting Information Figures S1d and e, the contours were not elliptical, indicating that EPA and DHA and EPA and ALA had no significant interactions with MDA content. Supporting Information Figure S2 shows that the slope of each response surface was steep, indicating that interactions between EPA, ALA, and DHA had an impact on ROS levels, GST activity, and MDA content. The slope of EPA×ALA and DHA×ALA were steeper, which indicated that they had good interactions.

### Validation and determination of optimal n-3 PUFA concentrations for antioxidant effects

Minitab statistical software 21 was used to analyze the experimental equation. The optimal n-3 PUFA concentrations were 145.46 µM EPA, 405.05 µM ALA, and 551.52 µM DHA. Under the optimized conditions, the ROS level was 16.865%, the MDA content was 0.8418 nmol/mg protein, and the GST activity was 32.754 U/mg protein. In this work, three validation parallel tests were conducted, and the average ROS level, MDA content, and GST activity were 17.093%, 0.8537 nmol/mg protein, and 32.384 U/mg protein, respectively. The results were similar to the predicted values, which indicated that the formula optimization scheme obtained by the response surface method had high feasibility.

## Discussion

The primary objective of this study was to investigate the impact of various n-3 PUFAs on the antioxidant capabilities of NCTC 1469 cells and to ascertain the most effective concentration for this purpose. (1) The impact of different n-3 PUFA concentrations on cellular antioxidant capacity was determined by measuring intracellular ROS, the oxidation product MDA, and a variety of antioxidant enzymes. (2) The optimal concentration of n-3 PUFAs for cellular antioxidant effects was determined by a response surface optimization experiment. Some relevant studies on DHA and EPA concentrations were conducted previously, but this was the first study to investigate the optimal concentrations of DHA, EPA, and ALA together using the response surface optimization method. The findings are of considerable importance for the supplementation and application of n-3 PUFAs.

The research revealed that a suitable n-3 PUFA concentration could enhance cell viability, potentially because these fatty acids can modulate cell viability via multiple mechanisms. This modulation contributes to anti-apoptotic effects, such as the downregulation of caspase-3 expression, which, in turn, can decrease the incidence of apoptosis [32]. Excessive n-3 PUFAs are cytotoxic, which has a significant impact on the number and morphology of cells, inhibiting cell proliferation and causing cell death. This could be because n-3 PUFAs facilitate the accumulation of ROS, increasing mitochondrial calcium (Ca^2+^) levels and activating the c-Jun N-terminal kinase (JNK) pathway. These actions may lead to the opening of mitochondrial permeability transition pores, which can initiate the apoptosis process [33]. Cell treatment time has a significant effect on cell viability. Among the three treatment times, cell viability decreased the least, and the cell proliferation rate was high after 24 h of treatment. This might be due to the toxic accumulation of excessive cell growth products in the cell culture medium. These results provided a basis for selecting the concentrations used in the next experiment.

This study presented a divergence from the anticipated relationship between antioxidant responses and the number of double bonds within n-3 PUFA molecules. While a linear correlation might have been expected based on the premise that a higher number of double bonds could correlate with a higher propensity for pro-oxidative actions and, thus, a more robust antioxidant defense, the results indicated a more intricate dynamic. The variation could be linked to the distinct operational mechanisms of the various n-3 PUFAs, encompassing their unique metabolic routes, their particular structural functions within cellular membranes, and their specific influences on cellular signaling pathways. The changes in the measurement indexes are also discussed below.

The findings indicated that EPA, ALA, and DHA lowered the levels of ROS within cells, thereby alleviating oxidative stress. A previous study reported that the intracellular antioxidant defense system could be activated to combat the damage caused by excessive ROS [34]. Giordano et al. discovered that the addition of fish oil or n-3 PUFAs to the diet could decrease F2-isoprostaglandin concentrations [35]. F2-isoprostanes represent a group of compounds that resemble prostaglandin-F2 and are generated by the peroxidation of arachidonic acid in cell membranes, catalyzed by ROS. Consequently, n-3 PUFAs may substitute for arachidonic acid within the cell membrane, which can decrease the synthesis of F2-isoprostanes and contribute to mitigating oxidative stress. Richard et al. conducted a series of experiments proving that fatty acids could remove superoxides in an unsaturated bond-dependent manner [36]. Lagarde et al. observed that n-3 PUFA supplementation could induce oxidative effects once it surpassed a specific threshold. [37]. This may be because excessive n-3 PUFAs can inactivate intracellular enzymes, destroy cell membrane structures, produce excessive ROS, and restrict normal cell growth.

MDA, recognized as an oxidative stress biomarker, emerges as a final byproduct of lipid oxidation, a process initiated by the attack of ROS on the unsaturated fatty acids present in biofilm. Therefore, the MDA content serves as a reliable indicator of the extent of oxidative cell damage. The research results indicated that appropriate concentrations of EPA, ALA, and DHA could reduce the content of oxidation products and alleviate oxidative stress. Among them, ALA had the best effect, with the largest decrease in MDA, followed by EPA and DHA. This may be because EPA, ALA, or DHA can enhance related antioxidant enzymatic activity and reduce MDA levels within cells by activating endogenous antioxidant defense systems. Investigations have also revealed that EPA, ALA, and DHA interact with signal transduction molecules to improve oxidative stress and play an antioxidant role by affecting gene expression, thus achieving the purpose of preventing and treating diseases [38]. EPA, ALA, and DHA can also stimulate the antioxidant system of cells to produce the corresponding antioxidant substances, including vitamins, amino acids, and small molecular peptides, which are used to counteract free radical attacks on cells and reduce MDA levels. However, when the concentrations of EPA, ALA, and DHA are too high, the unsaturated bonds in the EPA, ALA, and DHA may be attacked by free radicals, which will induce tissue lipid peroxidation and increase MDA content.

As the main member of the second defense line in the antioxidant system, GST can catalyze covalent conjugation reactions between glutathione and toxic electrophilic and hydrophobic substances to produce substances with anti-lipid peroxidation effects. In addition, it can react with some exogenous or endogenous metabolites, such as cholic acid and hormones, which are insoluble in water, to generate some water-soluble compounds, and then they are excreted from the body, thereby protecting brain tissue from antioxidant damage and reducing cellular damage by free radicals and toxic substances [39]. The results showed that appropriate concentrations of EPA, ALA, or DHA could improve GST activity, thereby reducing oxidative damage and enhancing the antioxidant capacity. Among them, ALA had the best effect, with GST activity increasing the most, followed by EPA and DHA. This effect may have been caused by the increased expression of GSTM1 and GSTT1 polymorphic genes and the decreased expression of the tumor protein p53 gene, increasing GST activity and improving the antioxidant capacity of the body.

The study showed that DHA, ALA, and EPA could increase SOD and CAT activity to different degrees. The findings align with those of Li et al., which demonstrated that DHA increased the activity of SOD and CAT enzymes in a dosage-dependent manner [40]. Antioxidant enzymes constitute a vital part of the cell’s innate antioxidant defense mechanism. As a significant cellular antioxidant, SOD facilitates the breakdown of superoxide anion radicals into H_2_O_2_ and molecular oxygen (O_2_), a process critical for mitigating oxygen-related oxidative harm. CAT catalyzes the conversion of H_2_O_2_ to H_2_O and O_2_, thereby lessening the oxidative damage caused by H_2_O_2_. Both SOD and CAT are pivotal in neutralizing free radicals, diminishing oxidative stress, and bolstering the cells’ antioxidant capabilities.

In addition, EPA, ALA, and DHA can interact with receptors, which regulate various signal transduction pathways associated with ROS production, as well as the induction of antioxidant enzymes. Peroxisome proliferator-activated receptor-α (PPAR-α) is instrumental in modulating the transcriptional and proliferation of related liver peroxidase and zymosomal proliferator genes, while carnitine palmitoyltransferase1 (CPT-I) acts as a pivotal enzyme in β-oxidation within mitochondria. Research has indicated that high concentrations of ALA can activate the PPAR-α gene and then increase the expression level of the CPT-I gene in its target cells, thus promoting mitochondrial fatty acid oxidation [41]. Concurrently, Zgorzinska et al. discovered that DHA and EPA are capable of boosting antioxidant enzymatic activity, mitigating oxidative stress in astrocytes, and strengthening the antioxidant defense triggered by the activation of nuclear factor erythroid 2-related factor 2 (Nrf2) via the phosphoinositide 3-kinase-Akt (PI3K/Akt) signaling pathway. However, DHA is specifically implicated in the p38 mitogen-activated protein kinase (p38 MAPK) pathway-mediated accumulation of Nrf2 [25]. Thus, further research on the signal transduction pathway is needed.

n-3 PUFAs are recognized for their ability to enhance the cellular antioxidant capacity through several potential mechanisms. They may directly scavenge ROS, thereby reducing oxidative stress within cells, or modulate the activity of antioxidant enzymes, which are crucial for neutralizing ROS. Additionally, by integrating into cell membranes, n-3 PUFAs can increase the fluidity of cell membranes and potentially affect the performance of proteins embedded within the membrane that are integral to antioxidant defense mechanisms. They are also known to regulate gene expression related to the oxidative stress response, including genes that encode antioxidant enzymes. n-3 PUFAs can inhibit inflammatory pathways, improve mitochondrial function, and regulate cellular signaling pathways that affect the cellular response to oxidative stress. This is consistent with reports by Finocchiaro et al. and Ma et al. [42, 43]. Comprehending these processes is essential for leveraging the advantages of n-3 PUFAs to preserve cellular integrity and avert illnesses linked to oxidative stress.

The R^2^ values of the models showed that the actual and predicted values fit well and were consistent with the actual data [44]. The adjustment coefficient R^2^_Adj_ was used to adjust the model according to the variables and was used to predict the test results. The *P*-values of the three models indicated that the levels of the three models were extremely significant, and the equations were well-fitted. Moreover, the *P*-values of the missing fitting terms of the three models were not significant, which increased the reliability of the models. Consequently, these models can be utilized to ascertain the optimal concentrations with effects on ROS levels, MDA content, and GST activity.

Response surface slope steepness and the contour map can adequately reflect the influence of various factors on each index, and both slope steepness and contour shape can reflect the significance of each interaction. With increases in each factor, the results indicated that ROS levels and MDA content had minimum value points and GST activity had a maximum value. Therefore, the optimal concentration of EPA, ALA, and DHA could be obtained by designing the experiment according to the response surface.

In addition, when the optimal concentrations of the three n-3 PUFAs were combined, cellular ROS levels and MDA content were lower than when cells were treated with single n-3 PUFAs, and GST activity was higher, so the antioxidant capacity was stronger than that of single n-3 PUFAs. This suggests that the addition of EPA, ALA, and DHA at optimal concentrations might have better antioxidant effects than the n-3 PUFAs alone. The enhanced antioxidant response observed with combined treatment with EPA, ALA, and DHA at optimal ratios can be ascribed to several interrelated factors. The synergistic effects of these fatty acids allow for a more comprehensive defense mechanism against oxidative stress. When provided together, they can modulate multiple cellular pathways, potentially leading to a more balanced incorporation into cell membranes, which is crucial for maintaining membrane integrity and the function of membrane-bound proteins involved in antioxidant defenses [45]. This combined presence may also lead to a more effective regulation of gene expression related to antioxidant enzymes and anti-inflammatory responses, thus providing broader protection against ROS [46]. Additionally, the direct scavenging of reactive species by these n-3 PUFAs is likely to be more efficient when they are present at optimal ratios, complementing each other’s action and collectively reducing oxidative stress more effectively than any single n-3 PUFA. Furthermore, the inhibition of pro-oxidant pathways and the promotion of overall cellular redox balance are likely to be more pronounced with combined supplementation, culminating in significantly stronger antioxidant effects.

When discussing the metabolic synthesis of EPA and DHA from ALA, it is important to acknowledge the limited conversion rates in humans. Humans and animals can metabolize ALA to EPA and DHA, but the conversion is not highly effective. Additionally, DHA can be retro-converted to EPA under certain conditions, although this is also not a preferred metabolic pathway in the body. These conversions are significant because they highlight the necessity of obtaining n-3 PUFAs directly through dietary intake, as conversion from ALA may not meet the body’s requirements for these n-3 PUFAs.

### Strengths and limitations

In the study, the impact of three n-3 PUFAs, including DHA, EPA, and ALA, on the antioxidant capacity was investigated. The study innovatively used the response surface test method to obtain the optimal component concentration for antioxidant effects in mouse hepatocytes. However, this research had certain constraints. First, the mechanisms for antioxidant capacity were not exhaustively investigated. Therefore, the influence of the level of related genes on antioxidant capacity should be explored further through transcriptome sequencing and other methods. Second, the established regression equation model may not have included all of the variables and interactions that affect the antioxidant capacity, resulting in an incomplete analysis. Finally, animal experiments should be conducted to fully investigate the impact and mechanisms of n-3 PUFAs on antioxidant capacity.

## Conclusion

In summary, the effects of the concentrations of the main n-3 PUFAs, namely EPA, ALA, and DHA, on the antioxidant capacity of mouse hepatocytes were explored. Within a certain concentration range, EPA, ALA, and DHA increased the activity of intracellular antioxidant enzymes and decreased the content of MDA oxidation products and ROS levels, thereby increasing cellular antioxidant levels. A quadratic polynomial regression equation model was established by the response surface method. The optimal n-3 PUFA treatment concentrations for antioxidant effects were determined to be 145.46 µM EPA, 405.05 µM ALA, and 551.52 µM DHA, and their antioxidant effects were superior to treatment with single n-3 PUFAs. These findings could have implications for the prevention and treatment of liver diseases since liver function is integral to metabolism, detoxification, and immune responses. The modulation of antioxidant enzymes through n-3 PUFAs could contribute to a broader strategy for reducing oxidative stress-related pathologies. These findings lay a foundation for further explorations into the regulatory mechanism of n-3 PUFAs. Future research studies should delve into the long-term effects of these optimal concentrations on health, as well as their potential synergistic effects with other nutrients. Additionally, clinical trials are essential for validating the findings in mice and determining the appropriate consumption dosages and delivery methods. The results of this study could lead to personalized dietary recommendations for patients with compromised liver function or those at high risk of oxidative stress-related diseases. By integrating these n-3 PUFAs into therapeutic diets, healthcare providers may be able to improve patient outcomes by enhancing cellular antioxidant activity. The findings could inform public health initiatives aimed at promoting dietary habits that support liver health and overall well-being.

### Electronic supplementary material

Below is the link to the electronic supplementary material.

Supplementary Material 1

## Data Availability

No datasets were generated or analysed during the current study.
