# Supplementary material for: Optimization of concentrations of different n-3PUFAs on antioxidant capacity in mouse hepatocytes
Source: Lipids Health Dis. 2024 Jul 9;23:214. doi: 10.1186/s12944-024-02202-0 (PMC11232338; doi:10.1186/s12944-024-02202-0)
Supplement: Supplementary file 1 — Supplementary Material 1 [file 12944_2024_2202_MOESM1_ESM.docx]

**Supporting Information**

**Optimization of concentrations of different n-3PUFAs on antioxidant capacity in mouse hepatocytes**

Shuting Wang^1^, Huasong Bai^1^, Tong Liu^1^, Jiayi Yang^2^, Zhanzhong Wang^1^[[1]](#footnote-1)^*^

^1^Nourse Science Centre for Pet Nutrition, Wuhu 241200, China

^2^School of Chemical Engineering and Technology, Tianjin University, Tianjin 300072, China

**^*^**Corresponding author: wzz7698@tju.edu.cn

**Table S1.** Response surface design and results.

| Run | A EPA | B ALA | C DHA | Y_1_ ROS level (%) | Y_2_ MDA content (nmol/mgprot) | Y_3_ GST activity (U/mgprot) |
| --- | --- | --- | --- | --- | --- | --- |
| 1 | -1 | 0 | -1 | 23.86 | 1.181 | 27.31 |
| 2 | 0 | 0 | 0 | 16.95 | 0.872 | 32.88 |
| 3 | 1 | -1 | 0 | 27.05 | 1.416 | 24.94 |
| 4 | 1 | 0 | -1 | 23.16 | 1.098 | 27.92 |
| 5 | 0 | 0 | 0 | 16.37 | 0.765 | 33.25 |
| 6 | 0 | 1 | -1 | 28.97 | 1.578 | 23.95 |
| 7 | 0 | 1 | 1 | 29.51 | 1.659 | 23.92 |
| 8 | 1 | 0 | 1 | 24.25 | 1.227 | 27.06 |
| 9 | 0 | -1 | -1 | 30.59 | 1.740 | 22.56 |
| 10 | 1 | 1 | 0 | 26.47 | 1.337 | 25.09 |
| 11 | 0 | -1 | 1 | 29.93 | 1.698 | 22.85 |
| 12 | -1 | 0 | 1 | 22.59 | 1.024 | 28.81 |
| 13 | 0 | 0 | 0 | 17.28 | 0.896 | 32.06 |
| 14 | -1 | -1 | 0 | 27.83 | 1.465 | 24.26 |
| 15 | -1 | 1 | 0 | 25.99 | 1.314 | 26.35 |

**Table S2.** Variance analysis in a quadratic polynomial model of the ROS level(Y_1_).

| Variance term | Freedom | Adj SS | Adj MS | F-value | P-value | Significance |
| --- | --- | --- | --- | --- | --- | --- |
| Model | 9 | 315.2 | 35.02 | 275.3 | 0.000 | ** |
| A | 1 | 0.056 | 0.056 | 0.44 | 0.538 |  |
| B | 1 | 2.480 | 2.480 | 19.49 | 0.007 | ** |
| C | 1 | 0.012 | 0.012 | 0.09 | 0.773 |  |
| A^2^ | 1 | 12.49 | 12.49 | 98.21 | 0.000 | ** |
| B^2^ | 1 | 243.9 | 243.9 | 1917 | 0.000 | ** |
| C^2^ | 1 | 83.64 | 83.64 | 657.5 | 0.000 | ** |
| AB | 1 | 0.403 | 0.403 | 3.16 | 0.135 |  |
| AC | 1 | 1.390 | 1.390 | 10.93 | 0.021 | * |
| BC | 1 | 0.362 | 0.362 | 2.84 | 0.153 |  |
| Residual | 5 | 0.636 | 0.127 |  |  |  |
| Lack of Fit | 3 | 0.215 | 0.072 | 0.34 | 0.803 |  |
| Pure Error | 2 | 0.421 | 0.210 |  |  |  |
| Cor Total | 14 | 315.8 |  |  |  |  |

Significant at **P* < 0.05, ***P* < 0.01.

**Table S3.** Variance analysis in a quadratic polynomial model of the MDA content (Y_2_).

| Variance term | Freedom | Adj SS | Adj MS | F-value | P-value | Significance |
| --- | --- | --- | --- | --- | --- | --- |
| Model | 9 | 1.35292 | 0.15032 | 57.55 | 0.000 | ** |
| A | 1 | 0.00110 | 0.00110 | 0.42 | 0.544 |  |
| B | 1 | 0.02322 | 0.02322 | 8.89 | 0.031 | * |
| C | 1 | 0.00002 | 0.00002 | 0.01 | 0.942 |  |
| A^2^ | 1 | 0.00001 | 0.00001 | 0.00 | 0.966 |  |
| B^2^ | 1 | 1.06657 | 1.06657 | 408.35 | 0.000 | ** |
| C^2^ | 1 | 0.30404 | 0.30404 | 116.41 | 0.000 | ** |
| AB | 1 | 0.00130 | 0.00130 | 0.50 | 0.513 |  |
| AC | 1 | 0.02045 | 0.02045 | 7.83 | 0.038 | * |
| BC | 1 | 0.00378 | 0.00378 | 1.45 | 0.283 |  |
| Residual | 5 | 0.01306 | 0.00261 |  |  |  |
| Lack of Fit | 3 | 0.00333 | 0.00111 | 0.23 | 0.871 |  |
| Pure Error | 2 | 0.00973 | 0.00486 |  |  |  |
| Cor Total | 14 | 1.36598 |  |  |  |  |

Significant at **P* < 0.05, ***P* < 0.01.

**Table S4.** Variance analysis in a quadratic polynomial model of the GST activity (Y_3_).

| Variance term | Freedom | Adj SS | Adj MS | F-value | P-value | Significance |
| --- | --- | --- | --- | --- | --- | --- |
| Model | 9 | 174.014 | 19.335 | 121.08 | 0.000 | ** |
| A | 1 | 0.366 | 0.366 | 2.29 | 0.190 |  |
| B | 1 | 2.772 | 2.772 | 17.36 | 0.009 | ** |
| C | 1 | 0.099 | 0.099 | 0.62 | 0.467 |  |
| A^2^ | 1 | 8.951 | 8.951 | 56.05 | 0.001 | ** |
| B^2^ | 1 | 133.487 | 133.487 | 835.92 | 0.000 | ** |
| C^2^ | 1 | 42.638 | 42.638 | 267.01 | 0.000 | ** |
| AB | 1 | 0.943 | 0.943 | 5.90 | 0.059 |  |
| AC | 1 | 1.390 | 1.390 | 8.70 | 0.032 | * |
| BC | 1 | 0.024 | 0.024 | 0.15 | 0.713 |  |
| Residual | 5 | 0.798 | 0.160 |  |  |  |
| Lack of Fit | 3 | 0.061 | 0.020 | 0.06 | 0.979 |  |
| Pure Error | 2 | 0.737 | 0.369 |  |  |  |
| Cor Total | 14 | 174.812 |  |  |  |  |

Significant at **P* < 0.05, ***P* < 0.01.

**
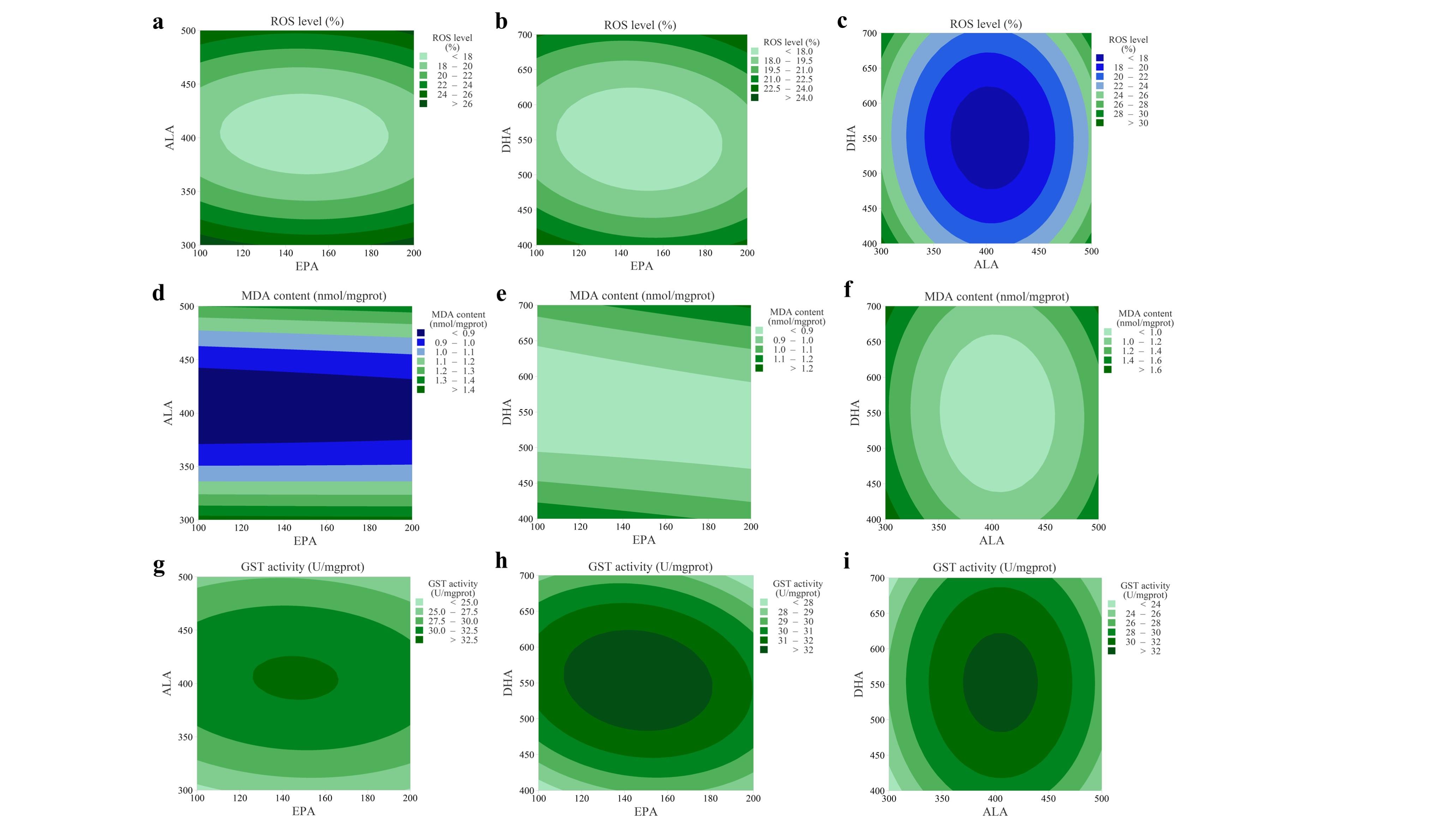
**

**Fig. S1** Contour plots of the interactions of n-3 PUFAs concentrations on the ROS level, MDA content and GST activity. Contour plots indicated the interaction between the factors. **a** shows the contour plot of the interaction of EPA and ALA on ROS level. **b** shows the contour plot of the interaction of EPA and DHA on ROS level. **c** shows the contour plot of the interaction of ALA and DHA on ROS level. **d** shows the contour plot of the interaction between EPA and ALA on MDA content. **e** shows the contour plot of the interaction between EPA and DHA on MDA content. **f** shows the contour plot of interaction between ALA and DHA on MDA content. **g** shows the contour plot of the interaction of EPA and ALA on GST activity. **h** shows the contour plot of the interaction of EPA and DHA on GST activity. **i** shows the contour plot of the interaction of ALA and DHA on GST activity. The results showed that EPA, ALA and DHA had significant interaction on ROS level and GST activity, while ALA and DHA had significant interaction on MDA content.

**
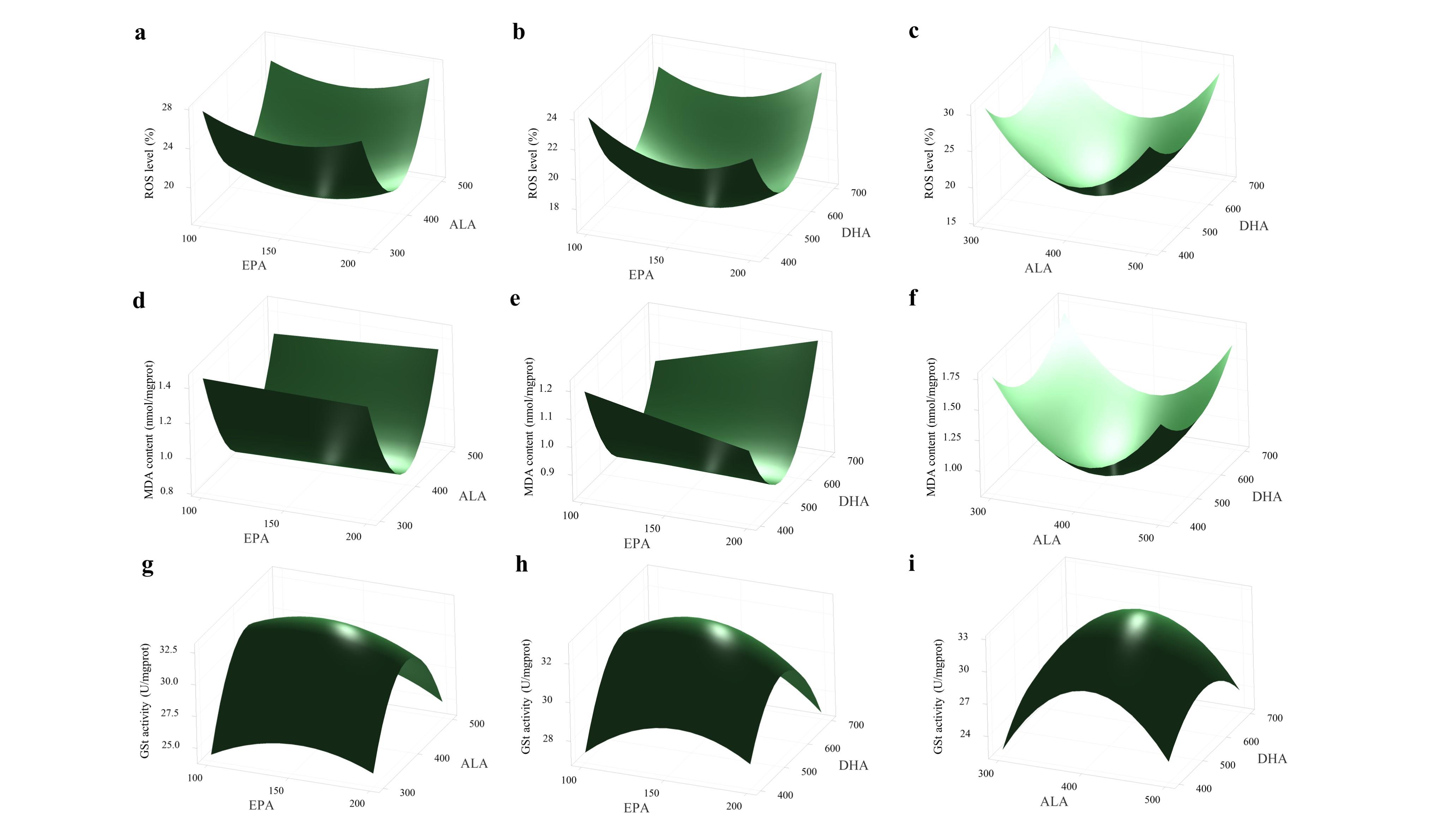
**

**Fig. S2** Response surface diagram of n-3 PUFAs concentrations on the ROS level, MDA content and GST activity. Response surface diagram indicated the interaction between the factors. **a** showed the response surface diagram of the interaction between EPA and ALA on ROS level. **b** showed the response surface diagram of the interaction between EPA and DHA on ROS level. **c** showed the response surface diagram of the interaction between ALA and DHA on ROS level. **d** showed the response surface diagram of the interaction between EPA and ALA on MDA content. **e** showed the response surface diagram of the interaction between EPA and DHA on MDA content. **f** showed the response surface diagram of the interaction between ALA and DHA on MDA content. **g** showed the response surface diagram of the interaction between EPA and ALA on GST activity. **h** showed the response surface diagram of the interaction between EPA and DHA on GST activity. **i** showed the response surface diagram of the interaction between ALA and DHA on GST activity. The results showed that the interaction of EPA, ALA and DHA had effects on ROS level, MDA content and GST activity.

1. *Corresponding author.

   *E-mail address*: [wzz7689@tju.edu.cn](mailto:wzz7689@tju.edu.cn) [↑](#footnote-ref-1)
